# Preoperative immunonutrition as a strategy for reducing oxidative stress and inflammation in pancreatic ductal adenocarcinoma

**DOI:** 10.3389/fimmu.2026.1889074

**Published:** 2026-08-24

**Authors:** Przemysław Kostro, Blanka Wolszczak-Biedrzycka, Edyta Kostro, Joanna Matowicka-Karna, Zbigniew Kamocki, Cezary Pawlukianiec, Mateusz Maciejczyk, Anna Zalewska, Justyna Dorf

**Affiliations:** 1 Department of Thoracic Surgery, Medical University of Bialystok, Bialystok, Poland; 2 Department of Psychology and Sociology of Health and Public Health, University of Warmia and Mazury in Olsztyn, Olsztyn, Poland; 3 Department of Conservative Dentistry, Medical University of Bialystok, Bialystok, Poland; 4 Department of Clinical Laboratory Diagnostics, Medical University of Bialystok, Bialystok, Poland; 5 2nd Clinical Department of General and Gastroenterological Surgery, Medical University of Bialystok, Bialystok, Poland; 6 Students Scientific Club “Biochemistry of Lifestyle Diseases” at the Department of Hygiene, Epidemiology and Environmental Health, Medical University of Bialystok, Bialystok, Poland; 7 Department of Hygiene, Epidemiology and Environmental Health, Medical University of Bialystok, Bialystok, Poland; 8 Experimental Dentistry Laboratory, Medical University of Bialystok, Bialystok, Poland

**Keywords:** biomarkers, cytokines, immunonutrition, oxidative stress, redox balance, pancreatic ductal adenocarcinoma

## Abstract

**Introduction:**

The aim of this study was to evaluate the effect of preoperative immunonutrition (IMN) on oxidative stress parameters and inflammatory cytokine concentrations in patients with pancreatic ductal adenocarcinoma (PDAC) undergoing surgical resection.

**Methods:**

The study included 52 patients with PDAC, who were divided into two groups: group 1 (n = 26), which received an immunonutrition formula (Impact Oral^®^) for seven days before surgery, and group 2 (n = 26), comprising patients who did not receive preoperative nutritional support. The control group consisted of 40 healthy individuals. Oxidative status parameters (Total Antioxidant Capacity -TAC, Total Oxidative Status - TOS, and Oxidative Stress Index -OSI, ferric reducing antioxidant power - FRAP), protein glycoxidation markers (Advanced Glycation End Products - AGEs, Advanced Oxidation Protein Products - AOPPs), lipid peroxidation markers (Malondialdehyde - MDA), and cytokine concentrations (IL1α, IL1β, IL6, IL10, and tumor necrosis factor - TNFα) were analyzed at three time points: upon admission, after seven days of nutritional intervention (before surgery), and on postoperative day 10.

**Results:**

Patients with PDAC exhibited significantly higher levels of oxidative stress and lower antioxidant potential than the control group. The redox balance was significantly improved, and the concentrations of pro-inflammatory cytokines (IL1α, IL1β, and IL6) decreased after seven days of immunonutrition. However, surgery led to a rapid increase in oxidative stress in both study groups.

**Discussion:**

Preoperative immunonutrition is an effective strategy for alleviating oxidative stress and the systemic inflammatory response in patients with PDAC, thereby improving preoperative preparation for surgical resection, which could play a key role in postoperative recovery in this patient population.

## Introduction

Pancreatic ductal adenocarcinoma (PDAC) is the most common malignant tumor of the pancreas that accounts for 85%–95% of all solid pancreatic tumors (1). According to the database published by Global Cancer Observatory (GLOBOCAN), pancreatic cancer was responsible for approximately 466,000 deaths worldwide in 2020, while 496,000 new cases were diagnosed. Consequently, pancreatic cancer was ranked as the 12^th^ most common cancer globally. In addition, GLOBOCAN predicts that the number of new pancreatic cancer cases will increase to 658,000 in 2030 and 844,000 in 2040, whereas the number of deaths is expected to rise to 621,000 and 801,000, respectively (2). Consequently, PDAC is considered one of the deadliest malignancies, with a median five-year survival rate of just 7.2% (3). This is largely because most patients present with unresectable, locally advanced, or metastatic disease at the time of diagnosis. Surgical resection combined with chemotherapy, including gemcitabine and FOLFIRINOX (a combination of oxaliplatin, irinotecan, fluorouracil, and leucovorin), has improved survival in patients with early-stage pancreatic cancer, but these treatment modalities remain insufficient in patients with late-stage disease (1). Moreover, surgical resection itself induces a significant oxidative and inflammatory response, leading to further depletion of antioxidants such as glutamine, arginine, vitamins A, C, and E, and omega-3 fatty acids, which adversely affects the patient’s condition. Both the carcinogenic process itself and the accompanying malnutrition, which affects approximately 85% of patients with pancreatic cancer, may trigger an abnormal host immune response and increase the risk of postoperative complications (4). Therefore, preoperative strategies should focus on reducing oxidative stress and improving immune system function through the implementation of immunomodulatory nutrition (IMN), also referred to as immunonutrition (IN) (5, 6). Preoperative immunonutrition, which includes preparations containing compounds with proven immunomodulatory and antioxidant properties, such as arginine, glutamine, omega-3 fatty acids, and nucleotides, has been shown to reduce postoperative complications, shorten hospital stay, and improve overall clinical outcomes in high-risk surgical patients. Arginine, one of the key components of immunonutrition, plays a central role in T-cell proliferation, nitric oxide synthesis, and wound healing (7). It has been shown to restore impaired cellular immunity commonly observed in cancer patients and to improve postoperative outcomes by reducing infectious complications. Omega-3 fatty acids, particularly eicosapentaenoic acid (EPA) and docosahexaenoic acid (DHA), exhibit anti-inflammatory properties by altering eicosanoid synthesis and decreasing the production of pro-inflammatory cytokines such as IL6 and TNFα (8). They also contribute to cell membrane stabilization and attenuation of oxidative stress by reducing lipid peroxidation (9). Nucleotides support rapid cellular turnover, particularly in tissues with high proliferative activity, such as the intestinal mucosa and immune cells. Nucleotide supplementation has been associated with enhanced antibody responses and improved macrophage function (10). Glutamine, although still debated in some clinical contexts, remains a crucial substrate for enterocytes and lymphocytes, and contributes to maintaining redox balance through its role in glutathione synthesis (11). Reduced glutathione (GSH) is one of the most important endogenous antioxidants, and its depletion in PDAC patients exacerbates oxidative stress and increases vulnerability to postoperative complications (12, 13).

Despite growing evidence supporting the benefits of immunonutrition in gastrointestinal oncology, its specific impact on oxidative stress in PDAC patients remains insufficiently elucidated. Several clinical trials and meta-analyses have demonstrated that perioperative immunonutrition can significantly reduce postoperative infections rates, shorten hospital stay, and improve immune parameters in patients undergoing upper gastrointestinal and pancreatic surgery. Although the strongest evidence concerns clinical outcomes, there is a growing interest in the mechanistic effects of immunonutrition on oxidative stress. By providing antioxidants and precursors for antioxidant pathways, immunonutrition may reduce the production of reactive oxygen species (ROS), thereby limiting oxidative damage to lipids, proteins, and DNA.

Given that oxidative stress plays a key role in surgical outcomes, tumor progression, and the systemic inflammatory response, evaluating the extent to which preoperative immunonutrition modulates oxidative biomarkers is of considerable importance. Patients with PDAC represent an especially vulnerable group due to high baseline oxidative imbalance, severe weight loss, and increased metabolic demand. Understanding whether immunonutrition can optimize their preoperative physiological status may contribute to improved postoperative recovery and potentially better long-term outcomes.

Therefore, the aim of this study was to evaluate the effect of preoperative immunonutrition on oxidative stress parameters and cytokine levels in patients with PDAC undergoing surgical treatment. Specifically, the study sought to determine whether targeted preoperative nutritional supplementation with immunomodulatory nutrients can reduce oxidative damage, improve antioxidant capacity, and modulate the perioperative inflammatory response in this high-risk patient population.

## Materials and methods

### Study design

The study included 52 patients (27 women and 25 men) aged 59 to 84 years, who underwent surgical resection for PDAC at the Second Department of General and Gastroenterological Surgery at the Clinical Hospital of the Medical University of Białystok (Poland) between 2020 and 2023. None of the patients had received preoperative radiotherapy or chemotherapy. Patients with coexisting malignancies were excluded from the study.

### Randomization

The patients were randomly assigned to two groups in a 1:1 ratio using a computer-generated randomization sequence. The randomization list was prepared before patient enrollment by Przemysław Kostro, MD, who was responsible for patient qualification and recruitment. Following confirmation of eligibility criteria and provision of written informed consent, patients were assigned to the immunonutrition group (Group 1) or the control group (Group 2) according to the predetermined randomization schedule. Group 1 (n = 26) consisted of patients with pancreatic cancer who received perioperative immunonutrition therapy (Impact Oral^®^), whereas group 2 (n = 26) comprised patients who did not receive nutritional support.

In group 1, blood was sampled at three time points: upon admission to the hospital, after seven days of immunonutrition, and on postoperative day 10. In group 2 (without nutritional treatment), blood samples were collected twice: upon admission to the hospital and on postoperative day 10.

At admission, nutritional status was assessed using the Subjective Global Assessment (SGA). Patients were classified according to the standard SGA categories: well nourished (SGA A), moderately malnourished or suspected of being malnourished (SGA B), and severely malnourished (SGA C). In addition, unintended preoperative weight loss was recorded and body mass index (BMI) was calculated. The following laboratory parameters were assessed: complete blood count with 5-part differential, C-reactive protein (CRP), cancer antigen 19-9 (CA19-9),carcinoembryonic antigen (CEA), total protein (TP), albumin, and total bilirubin.

### Immunonutrition protocol

Patients assigned to the immunonutrition group received Impact Oral^®^ under direct hospital supervision during the preoperative hospitalization period. The supplement was administered for seven consecutive days before surgery at a dose of one 237 mL package (300 kcal) three times daily. Compliance with the prescribed regimen was monitored by clinical staff, and the supplement was distributed daily according to the study protocol. All patients completed the prescribed immunonutrition regimen and received the recommended dose. No patients were excluded because of poor compliance or incomplete supplement consumption. No adverse events associated with immunonutrition were recorded.

### Blinding

Due to the nature of the nutritional intervention, blinding of patients, recruiting investigators, and surgeons was not feasible. Blood samples were anonymized and identified by unique study codes before laboratory analyses were performed. Consequently, laboratory personnel were blinded to group allocation. Statistical analyses were performed using coded datasets; however, no formal blinding of the statistician was implemented.

### Control group

The control group consisted of 40 healthy individuals who attended routine follow-up visits at the Specialist Dental Outpatient Clinic (Department of Conservative Dentistry) of the Medical University of Białystok between January 2020 and January 2021. The control group was matched to the study group in terms of age and sex. Individuals in the control group reported no use of medications or dietary supplements and denied cigarette smoking. They were in good general health, as confirmed by the absence of deviations from the reference values in complete blood count and biochemical parameters (Na+, K+, creatinine, INR, ALAT, and ASPAT), as well as the absence of comorbidities.

All participants provided written informed consent to participate in the study. The study protocol was approved by the Bioethics Committee of the Medical University of Białystok (decision No. APK.002.237.2020 of 25 June 2021).

### Biological material

The biological material for analysis consisted of serum samples obtained from both study groups and the control group. Venous blood was collected into S-Monovette^®^ tubes containing a clot activator (Sarstedt, Germany) and centrifuged at 1500 × g for 10 min at +4 °C (MPW 351, MPW Med. Instruments, Warsaw, Poland). Approximately 0.5 M butylated hydroxytoluene (20 µL per 2 mL of plasma or serum) was added to the serum to prevent sample oxidation. All samples were stored at a temperature of -80 °C until analysis.

### Oxidative status parameters: totalantioxidant capacity, total oxidative status,oxidative stress index and ferric reducing antioxidant power

TAC levels were determined using a colorimetric method (at a wavelength of 660 nm), based on the reaction between the 2,2’-azinobis-(3-ethylbenzothiazoline-6-sulfonic acid) (ABTS) radical cation and antioxidants present in the serum, and were calculated from a calibration curve prepared using 6-hydroxy-2,5,7,8-tetrametylchromane-2-carboxylic acid (Trolox) (14).

TOS levels were determined using a colorimetric method (at wavelengths of 560/800 nm), based on the oxidation of Fe^2+^ to Fe^3+^ in the presence of oxidants contained in the serum (15), and were expressed in micromolar hydrogen peroxide equivalents per liter.

The OSI was calculated using the following formula: OSI = (TOS/TAC) × 100%. Ferric reducing antioxidant power (FRAP) in the serum was determined using a colorimetric method (at a wavelength of 593 nm) with 2,4,6-tripyridyl-s-triazine (2,4,6-TPTZ). FRAP levels were calculated from a calibration curve prepared using FeSO_4_ (16).

### Parameters of oxidative protein damage: advanced glycation end products advanced oxidation protein products, and lipid peroxidation products: malondialdehyde

AGE levels were determined fluorometrically by measuring AGE-specific fluorescence at wavelengths of 250/440 nm in black-bottom 96-well microplates (90). Serum samples were diluted 1:50 (v/v) in phosphate-buffered saline (0.02 M, pH 7.0). The results were expressed in AFU/L (arbitrary fluorescence units per liter) (17).

AOPP concentrations were determined colorimetrically at a wavelength of 340 nm by measuring the oxidative capacity of iodide ions (90). Serum samples were diluted 1:50 (v/v) in phosphate-buffered saline (0.02 M, pH 7.0). The results were expressed in μmol/L (17).

MDA levels were determined colorimetrically at a wavelength of 326 nm using the thiobarbituric acid reactive substances (TBARS) method (91). 1,3,3,3-tetraethoxypropane was used as the standard (18). The results were expressed in mg/L.

### Cytokine detection

The concentrations of IL1α, IL1β, IL6, IL10, and TNFα were determined using the Cytokine Screening Panel, 48-Plex (#12007283). The Bio-Plex technology is a multiplex ELISA assay based on magnetic beads. In this test, captured antibodies directed against specific biomarkers are covalently coupled to magnetic beads. The conjugated beads are then incubated with samples containing selected biomarkers. Unbound proteins are removed through a series of washing steps, after which a biotinylated detection antibody is added to form a sandwich complex. The final complex is generated by adding a streptavidin-phycoerythrin (SA-PE) conjugate. Readings are carried out using a dedicated plate reader (Bio-Plex 200), and the performance of this method is comparable to that of a conventional ELISA assay.

### Statistical analysis

Statistical analysis was performed using GraphPad Prism 10.0 software. The normality of data distribution was assessed using the Shapiro-Wilk test. Comparisons between two independent groups were performed using Student’s t-test for normally distributed variables and the Mann-Whitney U test for non-normally distributed variables. Comparisons within the same group were performed using the Wilcoxon signed-rank test.

Comparisons among three independent groups (control group, PDAC patients receiving immunonutrition, and PDAC patients without immunonutrition) were performed using one-way analysis of variance (ANOVA) followed by Tukey’s multiple comparisons test for normally distributed variables. For non-normally distributed variables, the Kruskal-Wallis test followed by Dunn’s multiple comparisons test was applied.

Results were presented as median values (minimum–maximum). A two-sided p-value < 0.05 was considered statistically significant.

No formal adjustment for multiple comparisons across all study outcomes was performed. Given the exploratory nature of the study and the number of statistical tests conducted, the results should be interpreted with caution due to the increased risk of Type I error associated with multiple testing.

## Results

### Characteristics of the study population

The study population comprised 27 women (52%) and 25 men (48%) aged 59 to 84 years. As many as 42.9% of the participants reported unintentional body weight loss exceeding 10% during the preceding three months, while 38.1% had lost more than 10 kg of body weight. The patients’ BMI ranged from 19.3 to 33.9 kg/m^2^, with a median value of 24.95 kg/m^2^ (**Table 1**, **Table 2**).

**Table 1:** Demographic and clinical characteristics of patients with pancreatic ductal adenocarcinoma (PDAC).

Parameter	Study population	Patients with immunonutrition	Patients without immunonutrition	P value
Number of patients	52	26	26
Age, in years				
<65	17 (31.0%)	5 (16.7%)	12 (45.8%)	p=0.007
≥65	35 (69.0%)	21 (83.3%)	14 (54.2%)	
Sex				
Male	25 (48%)	13 (50.0%)	12 (54.2%)	p=1.000
Female	27 (52%)	13 (50.0%)	14 (45.8%)	
Tumor location				
Head of pancreas	29 (57.1%)	16 (33.3%)	13 (50.0%)	p=0.577
Body and tail of pancreas	23 (42.9%)	10 (66.7%)	13 (50.0%)	
BMI, kg/m^2^				
18.5 – 24.9	28 (52.4%)	14 (50.0%)	14 (50.0%)	p=0.108
25.0 – 29.9	15 (31.7%)	5 (22.2%)	10 (41.7%)	
30.0 – 34.9	9 (15.9%)	7 (27.8%)	2 (8.3%)	
Unintended body weight loss				
< 10%	29 (57.1%)	16 (67.7%)	13 (50.0%)	p=0.458
≥ 10%	23 (42.9%)	10 (33.3%)	13 (50.0%)	
Body weight loss				
< 10 kg	32 (61.9%)	11 (44.4%)	21 (75.0%)	p=0.010
≥ 10 kg	20 (38.1%)	15 (55.6%)	5 (25.0%)	
Diabetes mellitus	14 (28.6%)	4 (16.7%)	10 (26.7%)	p=0.116
Jaundice caused by biliary stricture	17 (32.0%)	7 (27%)	10 (38%)	p=0.555
Preoperative endoscopic retrograde cholangiopancreatography (ERCP)	18 (35.7%)	11 (44.4%)	7 (29.2%)	p=0.382
Tumor size				
< 3 cm	24 (45.2%)	6 (22.2%)	18 (62.5%)	p=0.002
≥ 3 cm	28 (54.8%)	20 (77.8%)	8 (37.5%)	
Histological grade				
G1	4 (4.8%)	4 (11.1%)	0 (0.0%)	p=0.001
G2	40 (78.6%)	22 (88.9%)	18 (70.8%)	
G3	8 (16.6%)	0 (0.0%)	8 (29.2%)	
Primary tumor stage (pT)				
T1+T2	13 (23.8%)	6 (22.2%)	7 (25.0%)	p=0.986
T3+T4	39 (76.2%)	20 (77.8%)	19 (75%)	
Lymph node metastasis (pN)				
absent (N0)	28 (54.8%)	12 (44.4%)	16 (62.5%)	p=0.404
present (N1)	24 (45.2%)	14 (55.6%)	10 (37.5%)	
Distant metastasis (pM)				
absent (M0)	33 (64.3%)	18 (72.2%)	15 (58.3%)	p=0.565
present (M1)	19 (35.7%)	8 (27.8%)	11 (41.7%)	
Vascular invasion				
absent	5 (9.5%)	3 (11.1%)	2 (8.3%)	p=0.965
present	47 (90.5%)	23 (88.9%)	24 (91.7%)	

Data are presented as count and percentage, n (%). Categorical variables were compared between groups using the Pearson Chi-Square test or Fisher’s exact test where appropriate. Statistically significant p-values (p < 0.05) are indicated in bold. BMI, Body Mass Index; ERCP, Endoscopic Retrograde Cholangiopancreatography.

**Table 2:** Laboratory test results of patients with pancreatic ductal adenocarcinoma (PDAC).

Laboratory test results	Study population n=52	Patients with immunonutrition n=26	Patients without immunonutrition n=26
WBC (x10^3^/μL)	7.35 (2.95-18.34)	7.77 (4.58-18.34)	7.02 (2.95-12.72)
Neutrophil (x10^3^/μL)	4.54 (1.27-14.69)	5. 16 (2.12-14.69)	4.02 (1.27-8.45)
Lymphocyte (x10^3^/μL)	2.053 (0.54-8)	1.961 (0.86-8)	2.13 (0.54-8)
RBC (x10^6^/μL)	4.08 (2.41-5.32)	4.10 (3.24-4.92)	4.03 (2.41-5.32)
HGB (g/dL)	12.39 (7.6-12.39)	12.02 (9.5-14.3)	12.67 (7.6-17.2)
HCT (%)	36.52 (22.5-46.9)	36.03 (28.3-42.4)	36.91 (22.5-46.9)
PLT (x10^3^/μL)	258.2 (82-562)	263.21 (148-562)	194.3 (82-313)
CEA (ng/mL)	4.9 (1.5-39.5)	6.32 (1.7-39.5)	2.06 (1.5-3.7)
CA19-9 (U/mL)	198.2 (2.1-1200)	3.4 (2.1-399)	447.84 (5.4-1200)
CRP (mg/L)	29.4 (0-325.8)	25.09 (1-180)	32.95 (0-325.8)
Totalprotein	6.478 (5.3-7.6)	6.51 (5.3-7.6)	6.45 (5.7-7.4)
Albumin	3.282 (2.11-4.28)	3.18 (2.11-3.99)	3.36 (2.98-4.28)
Total bilirubin	3.259 (0.32-11.74)	3.19 (0.32-11.74)	3.32 (0.49-10.21)

Data are presented as the median (min - max) of parameters. CA19-9, cancer antigen 19-9; CEA, carcinoembryonic antigen; CRP, C-reactive protein; HCT, Hematocrit; HGB, Hemoglobin; PLT, Platelets; RBC, Red blood count; WBC, White blood count.

### Group 1: patients receiving the impact oral^®^ immunomodulatory nutrition formula

This group consisted of 13 women and 13 men aged 59 to 83 years. The median age was 69 years. Sixteen patients had pancreatic head cancer, while the remaining 10 patients had tumors in the body and tail of the pancreas. In this group, BMI ranged from 21.2 to 33.9 kg/m^2^. Preoperatively, diabetes mellitus was diagnosed in four patients. Eleven patients had undergone endoscopic retrograde cholangiopancreatography (ERCP) with biliary stenting. Ten patients reported unintentional body weight loss exceeding 10% during the preceding three months.

### Group 2: patients who did not receive preoperative immunonutrition

This group comprised 14 women and 12 men aged 54 to 84 years. The median age was 64.5 years. Thirteen patients had pancreatic head cancer, while the remaining 13 patients had tumors in the body and tail of the pancreas. In this group, BMI ranged from 19.3 to 30.1 kg/m^2^.

### Comparison of preoperative TAC, TOS, OSI, and FRAP levels in patients with PDAC with immunonutrition and without immunonutrition and control group

**Figure 1:**
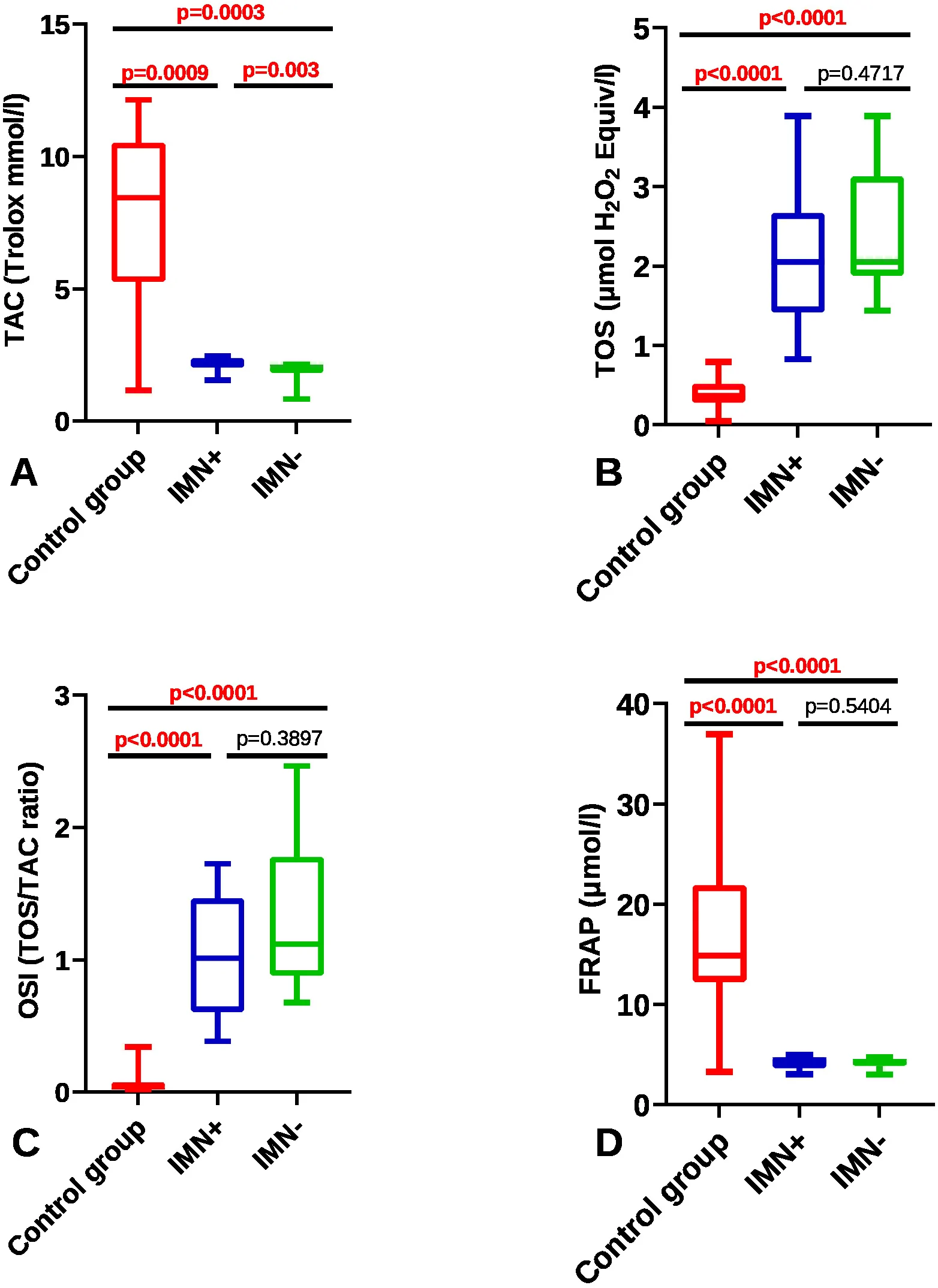
Comparison of preoperative TAC **(A)**, TOS **(B)**, OSI **(C)**, and FRAP **(D)** levels in patients with PDAC receiving (IMN+) and not receiving immunonutrition (IMN-), and in the control group. Data are presented as median values (minimum - maximum). TAC, total antioxidant capacity; TOS, total oxidant status; OSI, oxidative stress index; FRAP, ferric reducing antioxidant power; PDAC, pancreatic ductal adenocarcinoma.

In groups with immunonutrition and without immunonutrition patients, preoperative TAC (p<0.001) and FRAP (p<0.0001) levels were significantly lower, whereas TOS and OSI values were significantly higher than in the control group (p<0.0001). Among the analyzed parameters, only TAC levels differed significantly between groups 1 and 2, reaching higher values in the group of patients receiving immunonutrition than in patients without immunonutrition (p < 0.01) (**Figures 1A–D**).

### Comparison of preoperative AGE, AOPP, and MDA levels in patients with PDAC with immunonutrition and without immunonutrition and control group

**Figure 2:**
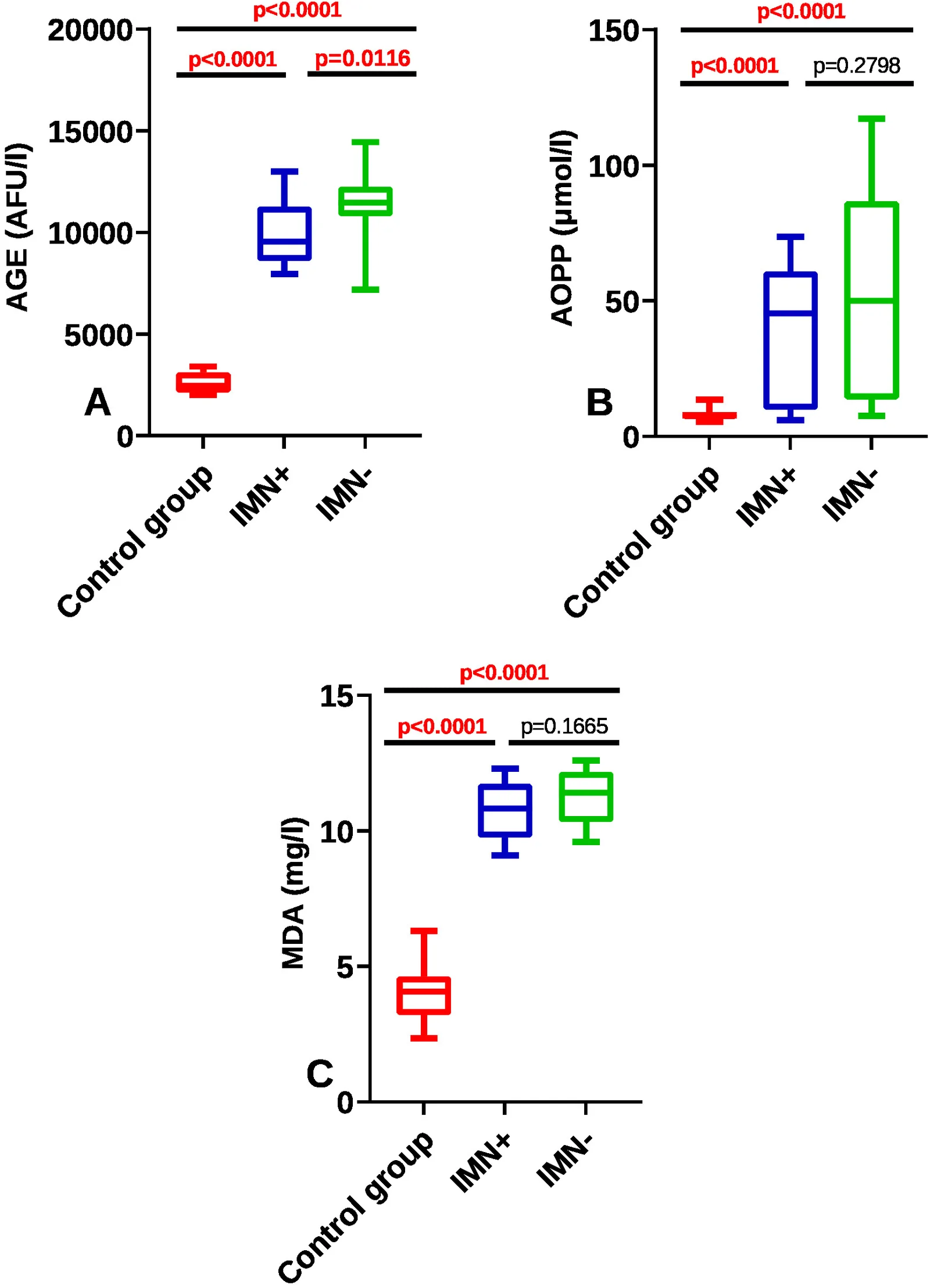
Comparison of preoperative AGE **(A)**, AOPP **(B)** and MDA **(C)** levels in patients with PDAC receiving (IMN+) and not receiving immunonutrition (IMN-), and in the control group. Data are presented as median values (minimum - maximum). AGE, advanced glycation end products; AOPP, advanced oxidation protein products; MDA, malondialdehyde; PDAC, pancreatic ductal adenocarcinoma.

Preoperative AGE, AOPP, and MDA levels were significantly higher in groups with immunonutrition and without immunonutrition patients than in the control group (p<0.0001) (**Figures 2A–C**). Among these parameters, only AGE levels differed significantly between groups IMN+ and 2, reaching lower values in patients receiving immunonutrition than in patients without immunonutrition (p < 0.05).

### Comparison of preoperative IL1α, IL1β, IL6, IL10, and TNFα levels in patients with PDAC with immunonutrition and without immunonutrition and control group

**Figure 3:**
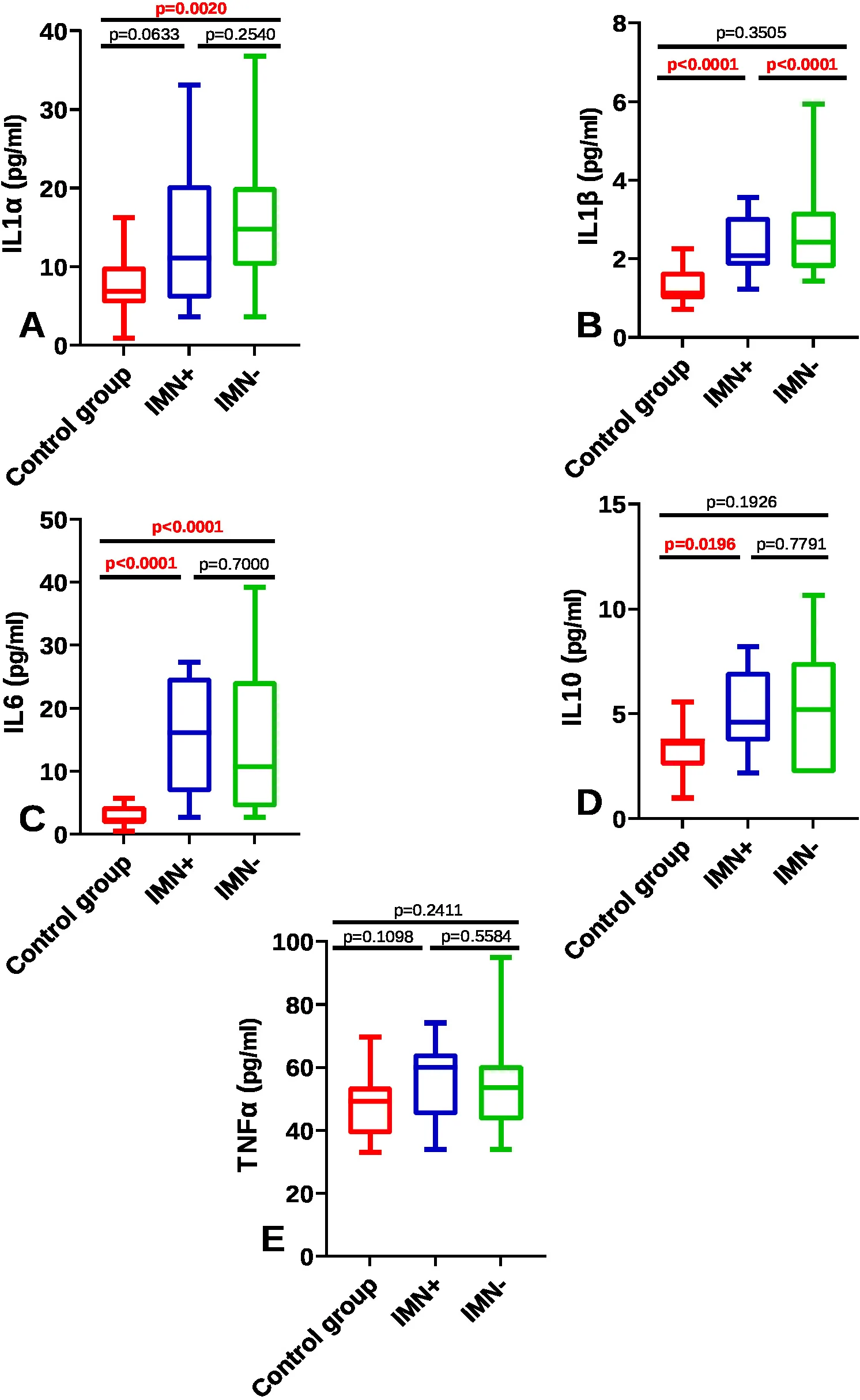
Comparison of preoperative inflammatory cytokine levels: IL1α **(A)**, IL1β **(B)**, IL6 **(C)**, IL10 **(D)**, and TNFα **(E)** in patients with PDAC receiving (IMN+) and not receiving immunonutrition (IMN-), and in the control group. Data are presented as median values (minimum - maximum). IL1α, interleukin-1 alpha; IL1β, interleukin-1 beta; IL6, interleukin 6; IL10, interleukin 10; TNFα, tumor necrosis factor alpha; PDAC, pancreatic ductal adenocarcinoma.

Preoperative levels of IL-1α (patients without immunonutrition), IL-1β (patients with immunonutrition), IL-6 (in both groups), and IL-10 (patients with immunonutrition) were significantly higher than those in the control group (*p* < 0.05; **Figures 3A–D**). Among these parameters, only IL-1β concentrations differed significantly between groups with immunonutrition and without immunonutrition, with lower values observed in the group with immunonutrition (*p* < 0.0001). Conversely, no significant differences were observed in TNF-α levels between the two groups (**Figure 3E**).

### Comparison of postoperative TAC, TOS, OSI, and FRAP Levels in patients with PDAC with immunonutrition and without immunonutrition and control group

**Figure 4:**
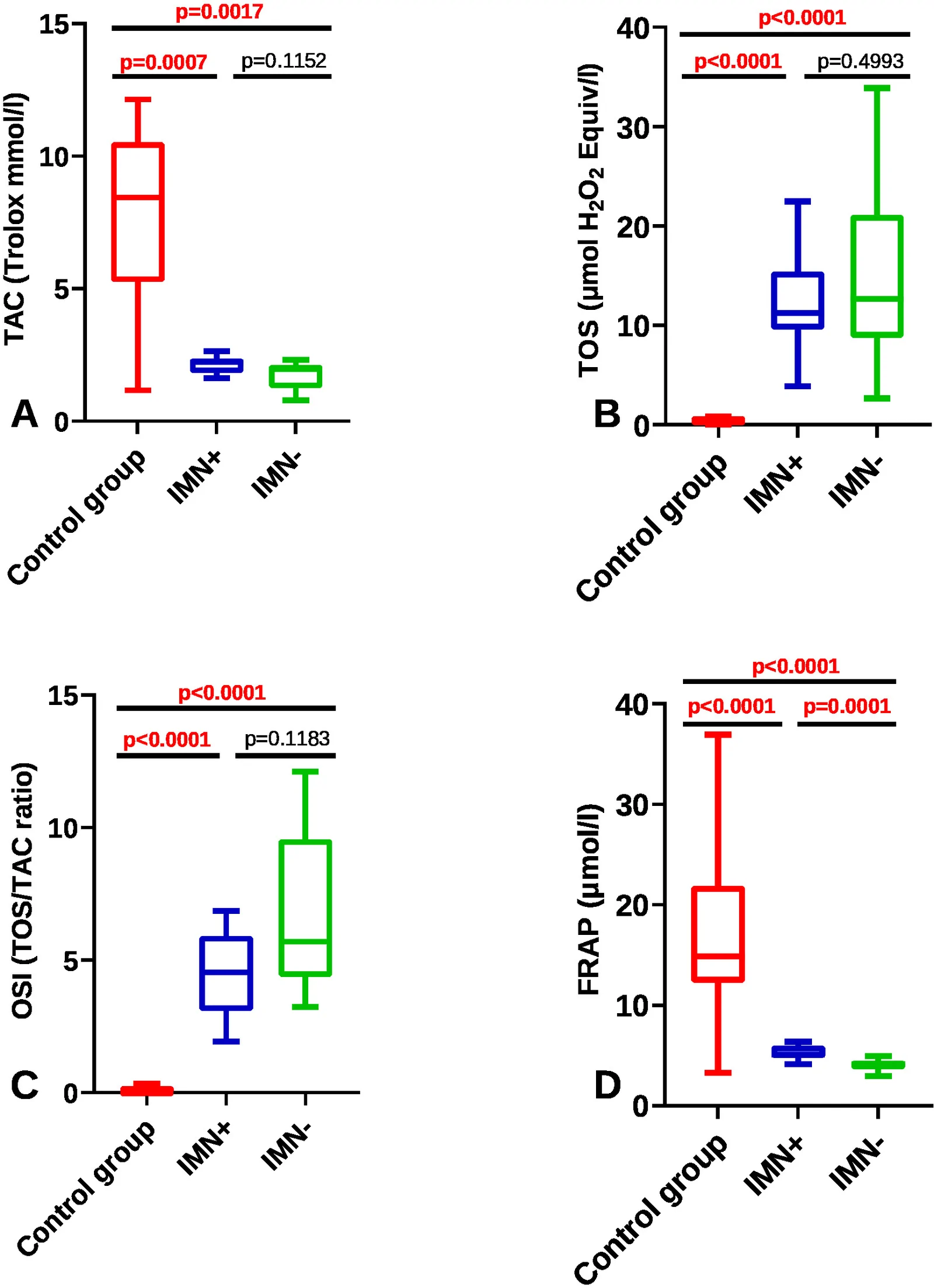
Comparison of postoperative TAC **(A)**, TOS **(B)**, OSI **(C)**, and FRAP **(D)** levels in patients with PDAC receiving (IMN+) and not receiving immunonutrition (IMN-), and in the control group. Data are presented as median values (minimum - maximum). TAC, total antioxidant capacity; TOS, total oxidant status; OSI, oxidative stress index; FRAP, ferric reducing antioxidant power; PDAC, pancreatic ductal adenocarcinoma.

Both PDAC groups showed significantly lower postoperative TAC and FRAP levels, as well as significantly higher TOS and OSI values compared with the control group (p<0.01; **Figures 4A–D**). Among these parameters, only FRAP levels differed significantly between groups 1 and 2, reaching higher values in group 1 (p < 0.0001).

### Comparison of postoperative AGE, AOPP, and MDA levels in patients with PDAC with immunonutrition and without immunonutrition and control group

**Figure 5:**
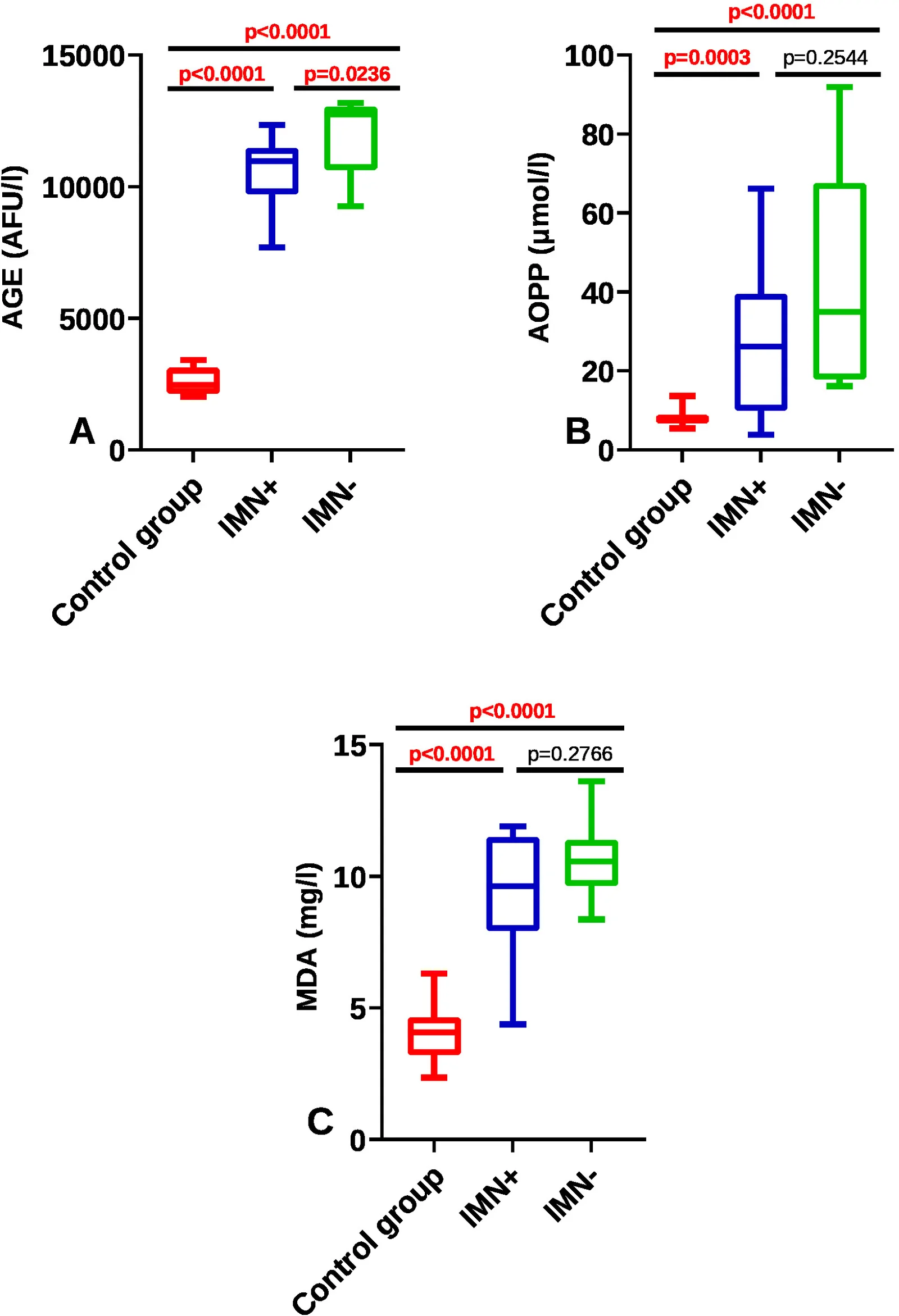
Comparison of postoperative AGE **(A)**, AOPP **(B)**, and MDA **(C)** levels in patients with PDAC receiving (IMN+) and not receiving immunonutrition (IMN-), and in the control group. Data are presented as median values (minimum - maximum). AGE, advanced glycation end products; AOPP, advanced oxidation protein products; MDA, malondialdehyde; PDAC, pancreatic ductal adenocarcinoma.

Postoperative AGE, AOPP, and MDA levels were significantly higher in groups 1 and 2 than in the control group (p<0.001; **Figures 5A–C**). Among these parameters, only AGE levels differed significantly between the two PDAC groups, reaching lower values in group 1 than in group 2 (p<0.05).

### Comparison of postoperative IL1α, IL1β, IL6, IL10, and TNFα levels in patients with PDAC with immunonutrition and without immunonutrition and control group

**Figure 6:**
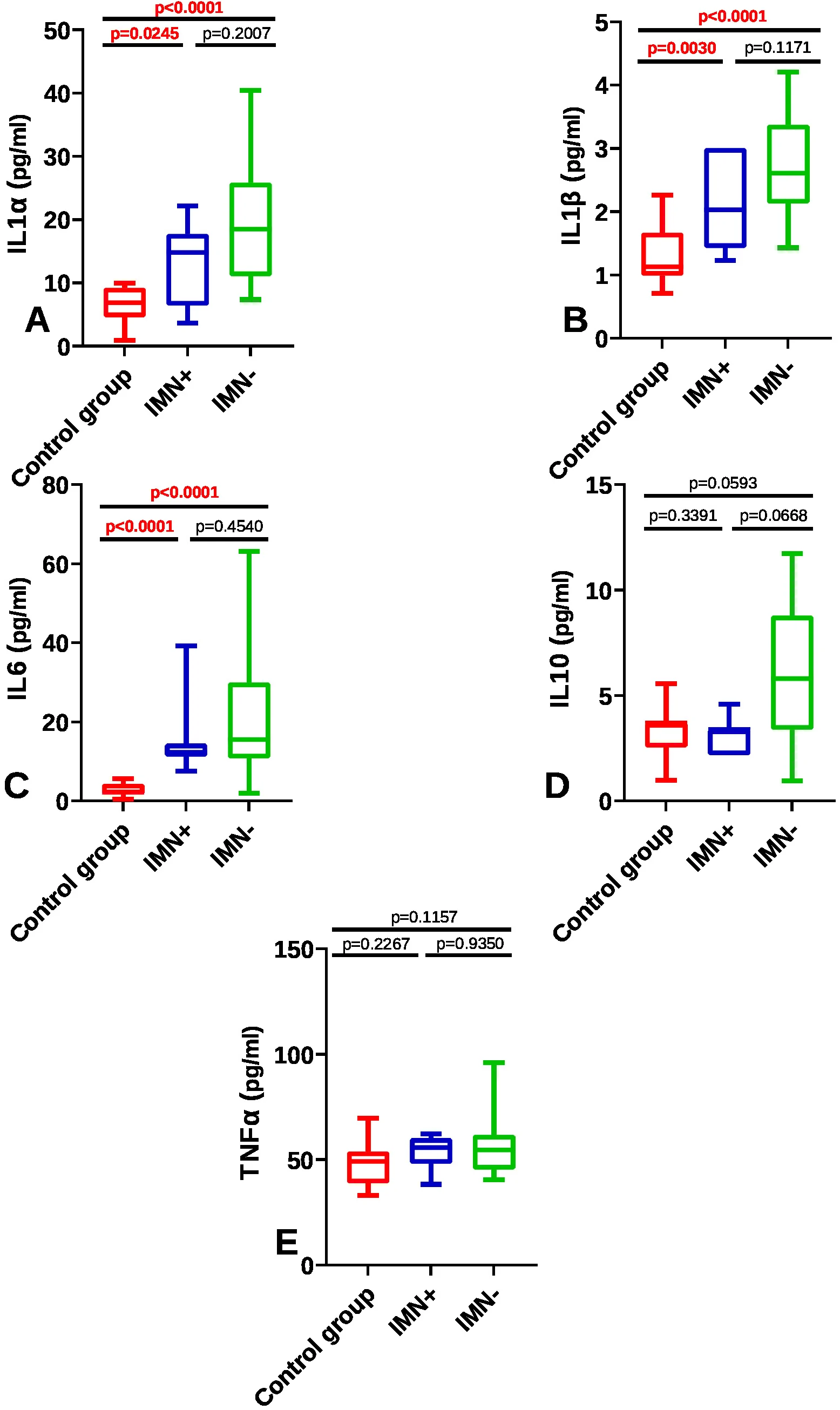
Comparison of postoperative inflammatory cytokine levels: IL1α **(A)**, IL1β **(B)**, IL6 **(C)**, IL10 **(D)**, and TNF-α **(E)** in patients with PDAC receiving (IMN+) and not receiving immunonutrition (IMN-), and in the control group. Data are presented as median values (minimum - maximum). IL1α, interleukin-1 alpha; IL1β, interleukin-1 beta; IL6, interleukin 6; IL10, interleukin 10; TNFα, tumor necrosis factor alpha; PDAC, pancreatic ductal adenocarcinoma.

Significantly higher postoperative IL1α, IL1β, and IL6 levels were observed in groups 1 and 2 than in the control group (p<0.01; **Figures 6A–C**). No significant differences were found in IL10 or TNFα concentrations (**Figures 6D, E**).

### TAC, TOS, OSI, and FRAP levels in patients with PDAC receiving immunonutrition, measured at admission (sample collection 1), after seven days of immunomodulatory nutritional treatment (sample collection 2), and on postoperative day 10 (sample collection 3)

**Figure 7:**
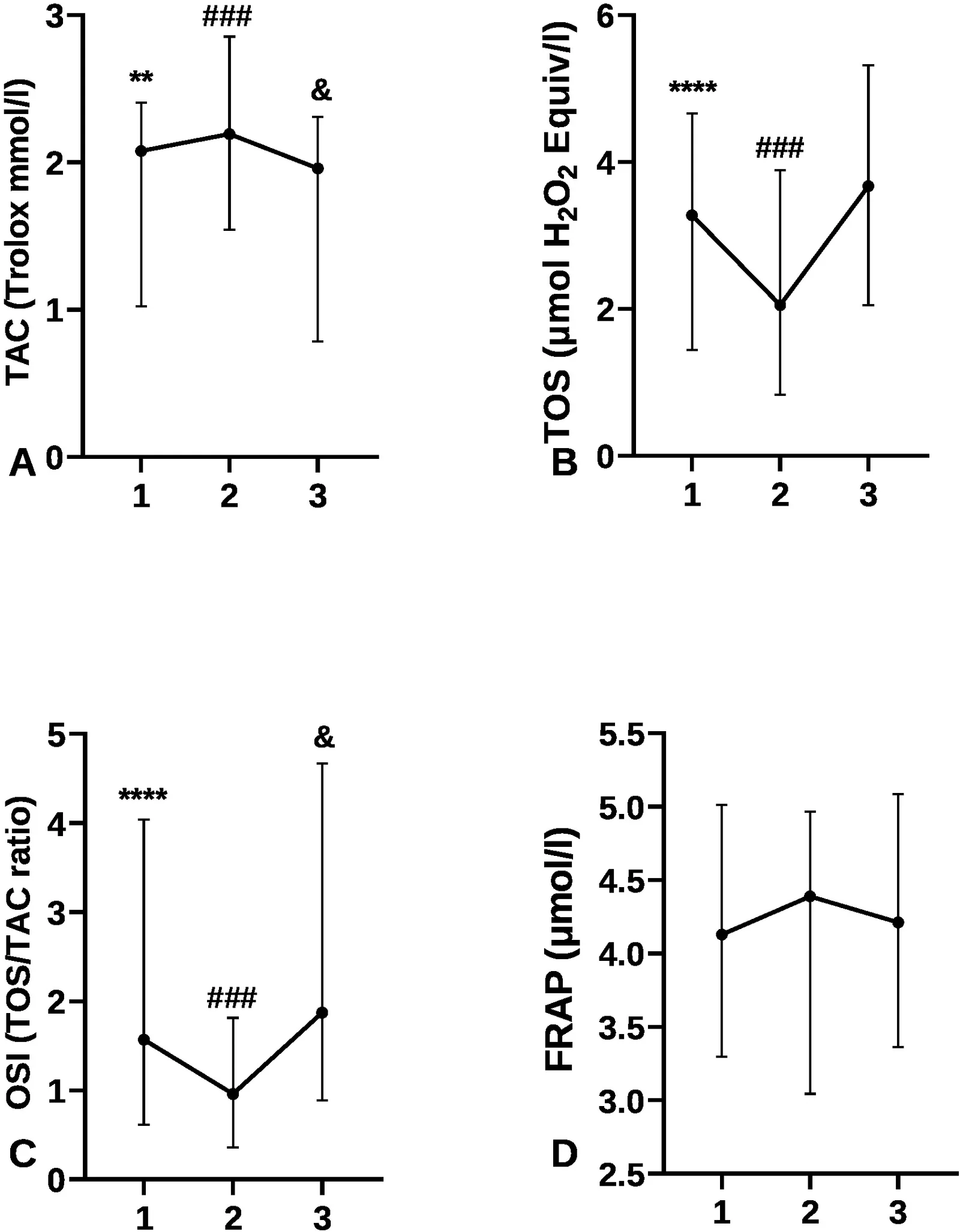
TAC **(A)**, TOS **(B)**, OSI **(C)**, and FRAP **(D)** levels in patients with PDAC receiving immunonutrition, measured upon hospital admission (1), after seven days of immunonutrition (2), and on postoperative day 10 (3). p < 0.05; **p < 0.01, ***p < 0.001, ****p < 0.0001 indicate significant differences between sample collections 1 and 2; ^#^p < 0.05, ^##^p < 0.01, ^###^p < 0.001, ^####^p < 0.0001 indicate significant differences between sample collections 2 and 3; ^&^p < 0.05, ^&&^p < 0.01, ^&&&^p < 0.001, ^&&&&^p < 0.0001 indicate significant differences between sample collections 1 and 3. TAC, total antioxidant capacity; TOS, total oxidant status; OSI, oxidative stress index; FRAP, ferric reducing antioxidant power; PDAC, pancreatic ductal adenocarcinoma.

After seven days of immunomodulatory nutritional treatment, redox parameters improved significantly in patients with PDAC, as reflected by increased TAC levels (p<0.01) and decreased TOS and OSI values (p<0.0001), compared with the baseline values before the initiation of nutritional treatment. On postoperative day 10, a subsequent increase in TOS and OSI values was observed relative to sample collection 1 (p<0.05) and sample collection 2 (p<0.001), whereas TAC levels were significantly lower than at sample collection 2 (p<0.001) and sample collection 1 (p<0.05) (**Figures 7A–C**). No statistically significant changes in FRAP levels were observed at any of the analyzed time points (**Figure 7D**).

### AGE, AOPP, and MDA levels in patients with PDAC receiving immunonutrition, measured at admission (sample collection 1), after seven days of immunomodulatory nutritional treatment (sample collection 2), and on postoperative day 10 (sample collection 3)

**Figure 8:**
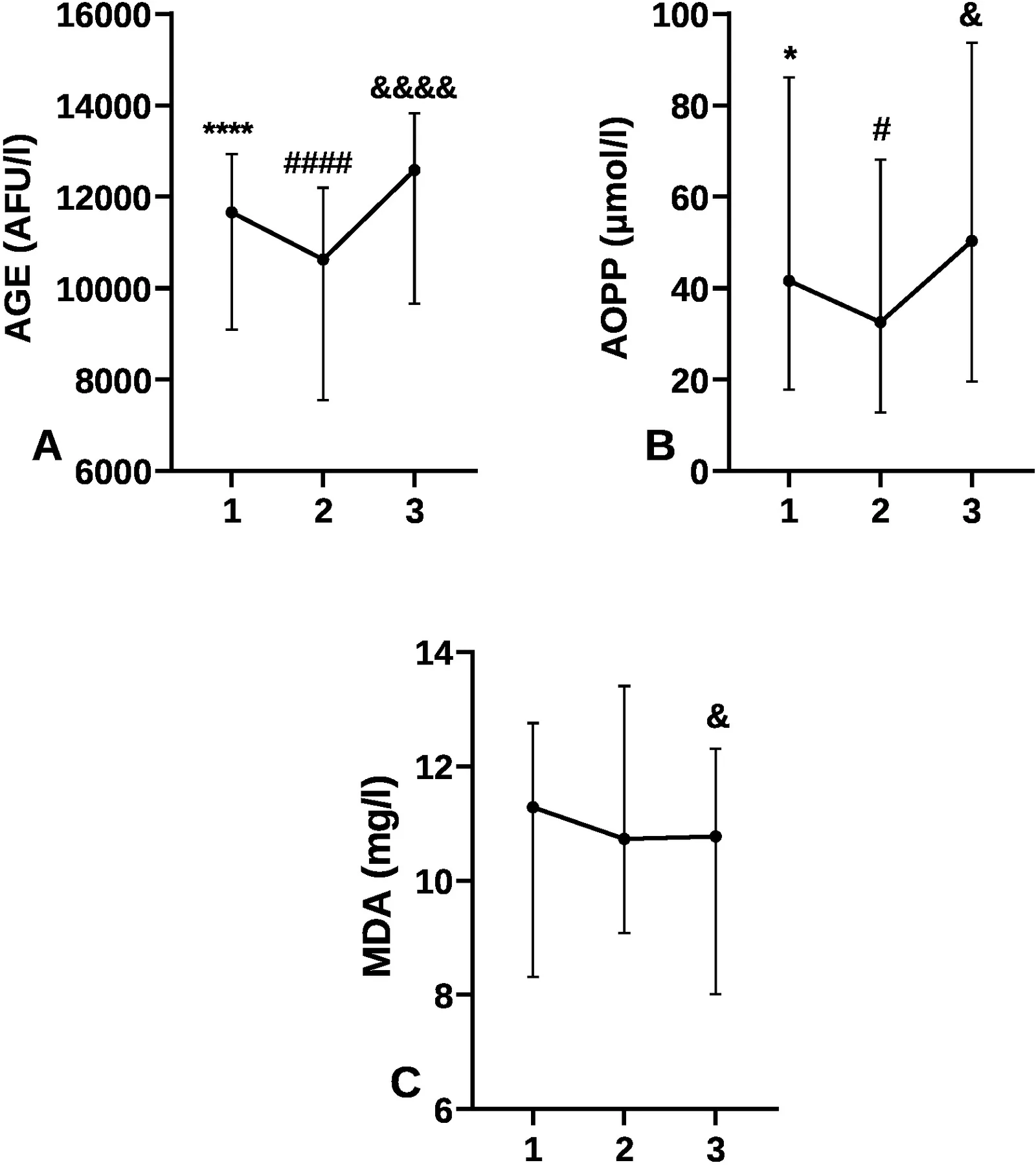
AGE **(A)**, AOPP **(B)**, and MDA **(C)** levels in patients with PDAC receiving immunonutrition, measured upon hospital admission (1), after seven days of immunonutrition (2), and on postoperative day 10 (3). *p < 0.05; **p < 0.01, ***p < 0.001, ****p < 0.0001 indicate significant differences between sample collections 1 and 2; ^#^p < 0.05, ^##^p < 0.01, ^###^p < 0.001, ^####^p < 0.0001 indicate significant differences between sample collections 2 and 3; ^&^p < 0.05, ^&&^p < 0.01, ^&&&^p < 0.001, ^&&&&^p < 0.0001 indicate significant differences between sample collections 1 and 3. AGE, advanced glycation end products; AOPP, advanced oxidation protein products; MDA, malondialdehyde; PDAC, pancreatic ductal adenocarcinoma.

After seven days of immunomodulatory nutritional treatment, a significant decrease in AGE (p<0.0001) and AOPP (p<0.05) levels was observed, compared with the baseline values before the initiation of treatment. However, on postoperative day 10, AGE (p<0.0001), AOPP (p<0.05), and MDA (p<0.05) levels increased significantly relative to the baseline values. In addition, AGE (p<0.0001) and AOPP (p<0.05) levels were significantly higher at sample collection 3 than at sample collection 2 (**Figures 8A–C**).

### IL1α, IL1β, IL6, IL10, and TNF-α levels in patients with PDAC receiving immunonutrition, measured at admission (sample collection 1), after seven days of immunomodulatory nutritional treatment (sample collection 2), and on postoperative day 10 (sample collection 3)

**Figure 9:**
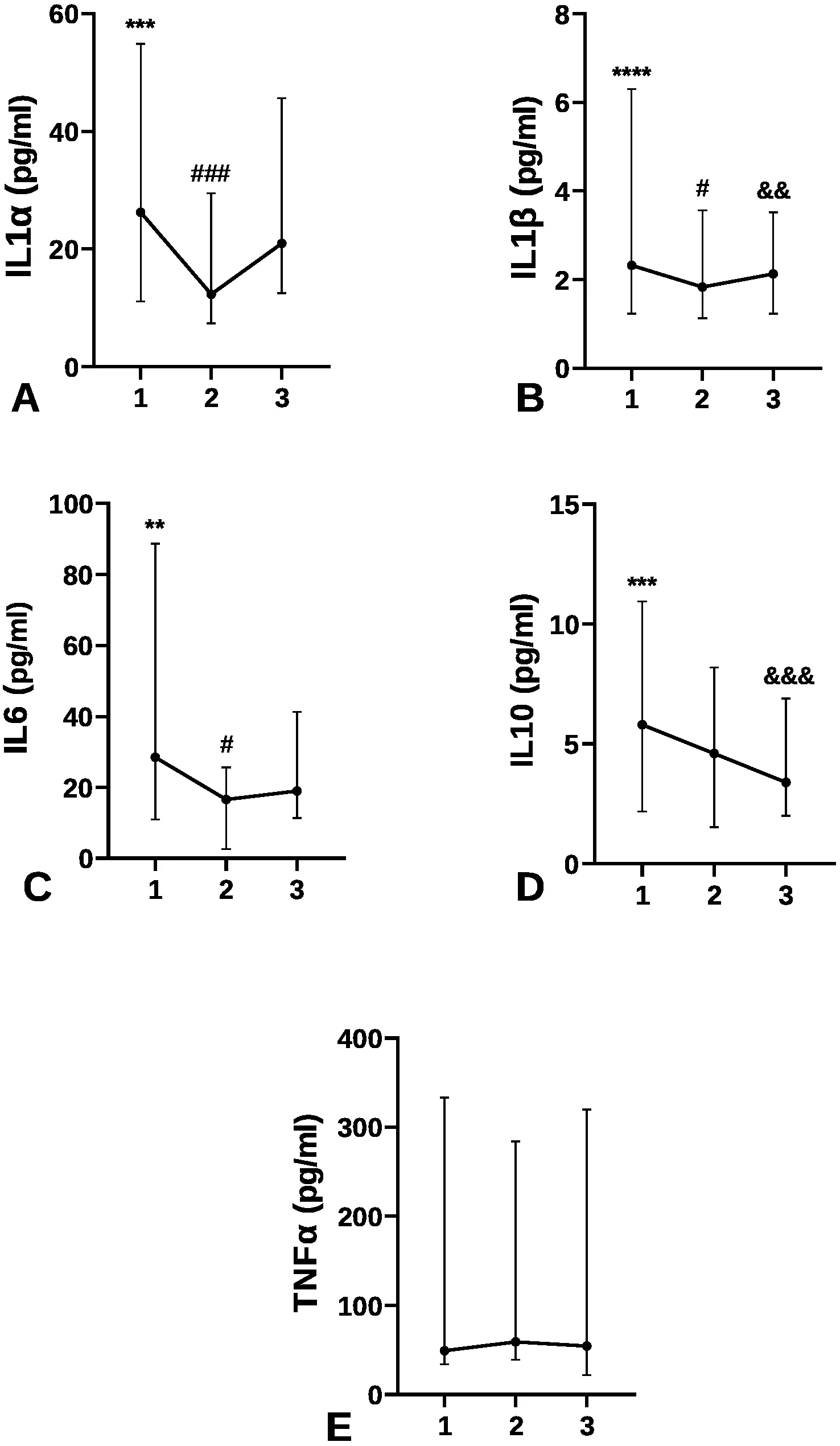
IL1α **(A)**, IL1β **(B)**, IL6 **(C)**, IL10 **(D)**, and TNFα **(E)** levels in patients with PDAC receiving immunonutrition, measured upon hospital admission (1), after seven days of immunonutrition (2), and on postoperative day 10 (3). p < 0.05; **p < 0.01, ***p < 0.001, ****p < 0.0001 indicate significant differences between sample collections 1 and 2; ^#^p < 0.05, ^##^p < 0.01, ^###^p < 0.001, ^####^p < 0.0001 indicate significant differences between sample collections 2 and 3; ^&^p < 0.05, ^&&^p < 0.01, ^&&&^p < 0.001, ^&&&&^p < 0.0001 indicate significant differences between sample collections 1 and 3. IL1α, interleukin1 alpha; IL1β, interleukin1 beta; IL6, interleukin 6; IL10, interleukin 10; TNFα, tumor necrosis factor alpha; PDAC, pancreatic ductal adenocarcinoma.

A significant decrease in IL1α (p<0.001), IL1β (p<0.0001), IL6 (p<0.05), and IL10 (p<0.001) levels was observed in PDAC patients after seven days of immunomodulatory nutritional treatment. On postoperative day 10, the concentrations of IL1α (p<0.001), IL1β (p<0.0001), and IL6 (p<0.05) increased again compared with the values observed at sample collection 2 (**Figures 9A–C**), whereas IL10 levels continued to decrease after surgery (p<0.001) (**Figures 9D, E**).

### TAC, TOS, OSI, and FRAP levels in PDAC patients without immunonutrition, measured at admission (sample collection 1) and on postoperative day 10 (sample collection 2)

**Figure 10:**
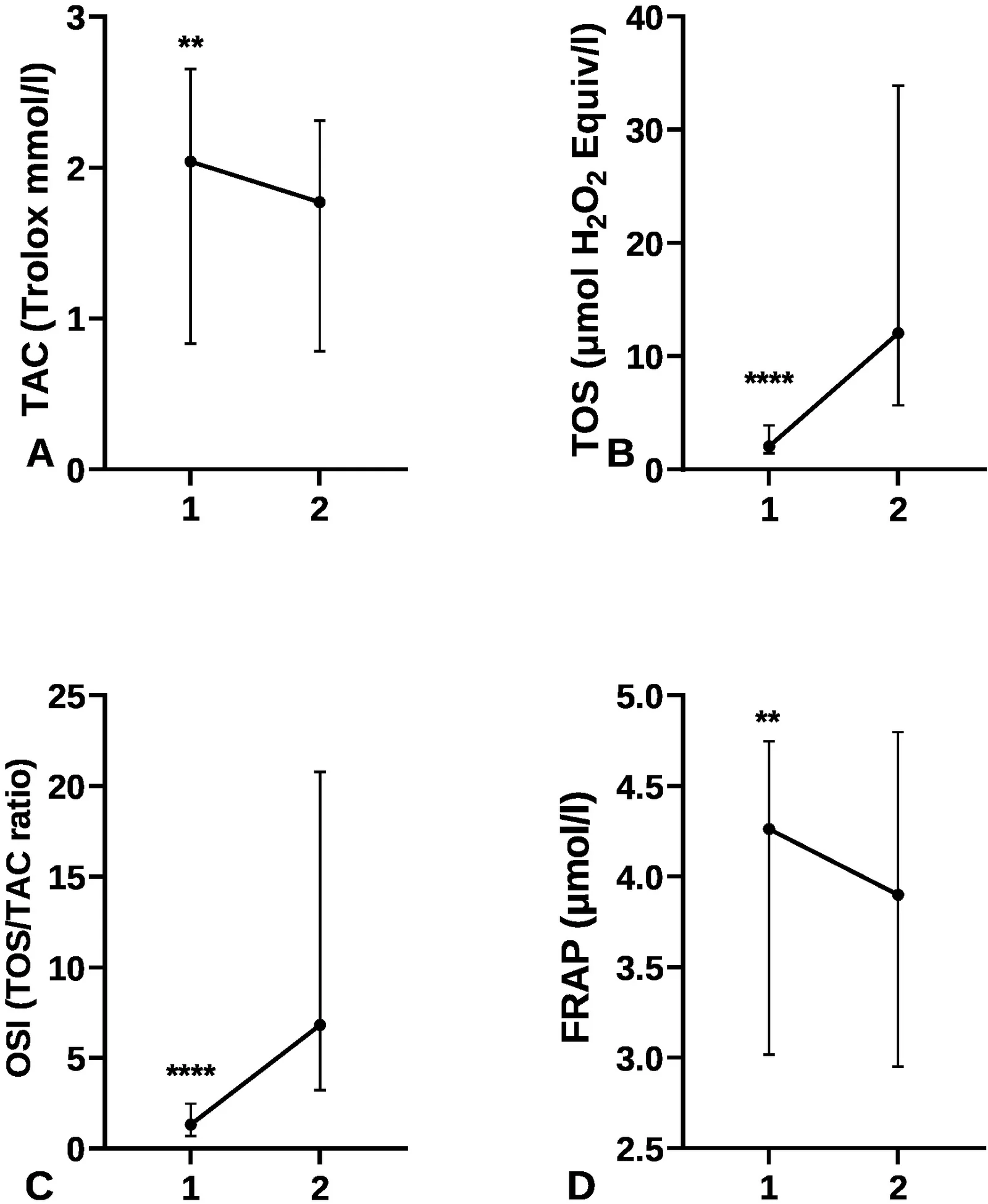
TAC **(A)**, TOS **(B)**, OSI **(C)**, and FRAP **(D)** levels in PDAC patients without immunonutrition, measured upon hospital admission (1) and on postoperative day 10 (2). p < 0.05; **p < 0.01, ***p < 0.001, ****p < 0.0001 indicate significant differences between sample collections 1 and 2. TAC, total antioxidant capacity; TOS, total oxidant status; OSI, oxidative stress index; FRAP, ferric reducing antioxidant power; PDAC, pancreatic ductal adenocarcinoma.

On postoperative day 10, a significant deterioration in redox parameters was observed, manifested by a decrease in TAC and FRAP levels (p<0.01) and a considerable increase in TOS and OSI values (p<0.0001), compared with the baseline values (**Figures 10A–D**).

### AGE, AOPP, MDA levels in PDAC patients without immunonutrition, measured at admission (sample collection 1) and on postoperative day 10 (sample collection 2)

**Figure 11:**
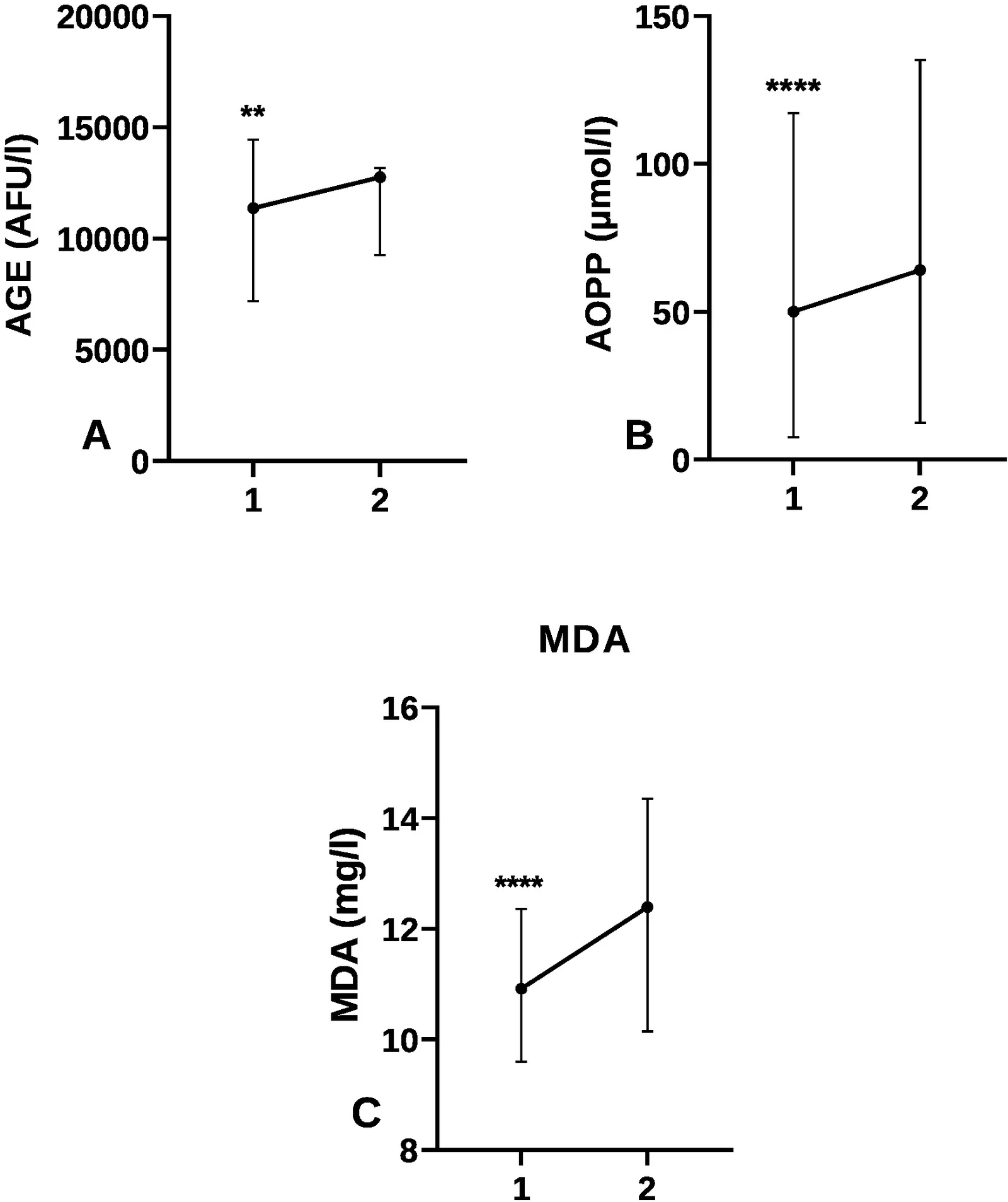
AGE **(A)**, AOPP **(B)**, and MDA **(C)** levels in PDAC patients without immunonutrition, measured upon hospital admission (1) and on postoperative day 10 (2). p < 0.05; **p < 0.01, ***p < 0.001, ****p < 0.0001 indicate significant differences between sample collections 1 and 2. AGE, advanced glycation end products; AOPP, advanced oxidation protein products; MDA, malondialdehyde; PDAC, pancreatic ductal adenocarcinoma.

On postoperative day 10, a statistically significant increase in AGE levels (p<0.01) as well as AOPP and MDA levels (p<0.0001) was observed relative to sample collection 1 (**Figures 11A–C**).

### IL1α, IL1β, IL6, IL10, and TNF-α levels in PDAC patients without immunonutrition, measured at admission (sample collection 1) and on postoperative day 10 (sample collection 2)

**Figure 12:**
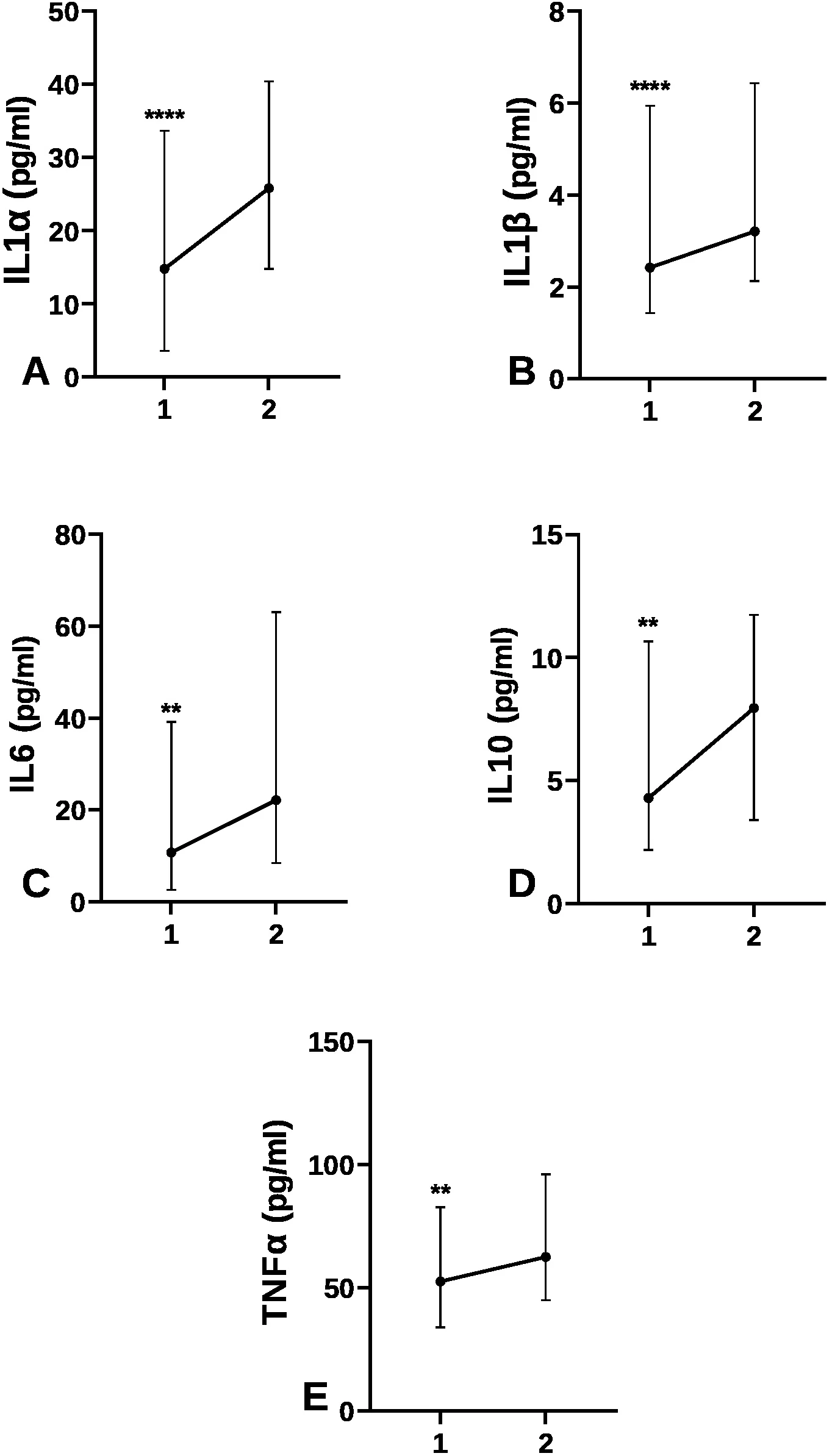
IL1α **(A)**, IL1β **(B)**, IL6 **(C)**, IL10 **(D)**, and TNFα **(E)** levels in PDAC patients without immunonutrition, measured upon hospital admission (1) and on postoperative day 10 (2). p < 0.05; **p < 0.01, ***p < 0.001, ****p < 0.0001 indicate significant differences between sample collections 1 and 2. IL1α, interleukin - 1 alpha; IL1β, interleukin-1 beta; IL6, interleukin 6; IL10, interleukin 10; TNFα, tumor necrosis factor alpha; PDAC, pancreatic ductal adenocarcinoma.

On postoperative day 10, a statistically significant increase in the concentrations of IL1α, IL1β (p<0.0001), IL6, IL10, and TNFα (p<0.01) was observed, relative to sample collection 1 (**Figures 12A–E**).

## Discussion

Oxidative stress plays a key role in the pathogenesis of many malignancies, including pancreatic cancer. Previous studies have shown that increased ROS production may cause damage to DNA, proteins, and lipids, thereby promoting tumor growth and progression (19, 20). In the present study, intensified oxidative stress was observed in patients with PDAC who received and did not receive immunomodulatory nutritional support, compared with the control group. A significant decrease in the antioxidant parameters TAC and FRAP was accompanied by significantly higher TOS and OSI (TOS/TAC ratio) values. Elevated OSI values in patients with pancreatic cancer suggest that oxidative processes predominate over antioxidant defense mechanisms. These findings confirm that pancreatic cancer is associated with impaired antioxidant response resulting from excessive ROS production (21). Enhanced oxidative stress in patients with pancreatic cancer has also been reported by other authors (22, 23). In the current study, the statistically significant decrease in antioxidant potential (TAC and FRAP) was accompanied by an increase in TOS, and this observation is consistent with previous reports demonstrating that the disruption of redox homeostasis is a characteristic feature of gastrointestinal malignancies and correlates with tumor stage (23, 24). Similarly elevated OSI values have also been reported in studies investigating gastrointestinal cancers, which identified OSI as a sensitive marker of metabolic imbalance in organisms affected by neoplastic disease (25).

Oxidative stress disrupts cellular metabolism through oxidative damage to cellular components such as proteins, lipids, and DNA. AGEs and AOPPs are among the most commonly assessed biomarkers of protein glycoxidation (26, 27). In the present study, AGE and AOPP levels were significantly higher in patients with PDAC than in the control group. AGEs can bind to receptors for advanced glycation end products (RAGEs), leading to the activation of pro-inflammatory signaling pathways. The above may promote chronic inflammation associated with tumor development and progression (28, 29). In addition, the accumulation of AGEs and AOPPs may affect the tumor microenvironment and promote angiogenesis and tumor growth. The present findings concerning elevated levels of protein damage markers AGEs and AOPPs are consistent with current research on the role of the tumor microenvironment (30, 31). Kodydkova et al. (32) reported higher concentrations of oxidative stress biomarkers and lower antioxidant levels in patients with pancreatic cancer than in patients with chronic pancreatitis, which suggests that intensified glycoxidation may be a useful indicator of malignant transformation in tissues. The observed correlation between increased AGE levels and potential activation of the RAGE pathway corroborates the findings of Zhang et al. (33), who demonstrated that the AGE-RAGE axis stimulates the secretion of pro-inflammatory cytokines, including IL6, and promotes epithelial-mesenchymal transition (EMT) in cancer cells, thereby facilitating metastasis. The elevated AOPP concentrations observed in PDAC patients in this study have been described in the literature as both a consequence of oxidative stress and an active factor that promotes neutrophil degranulation and further ROS production. These findings confirm that the antioxidant barrier is considerably weakened in pancreatic cancer and that accumulated protein glycoxidation products actively contribute to disease progression (34).

MDA is considered one of the most reliable and, at the same time, most harmful products of lipid peroxidation that contributes to altered gene expression, genetic mutations, and impaired intercellular communication (35). The accumulation of MDA-induced DNA damage accelerates malignant transformation by inducing replication errors and disrupting cell cycle control mechanisms (36). In addition, MDA plays an important role in the reprogramming of immune cells within the tumor microenvironment. It may directly influence macrophage polarization by shifting macrophages toward a phenotype that promotes tumor growth. Elevated MDA levels correlate with the recruitment of tumor-associated macrophages (TAMs), which secrete pro-inflammatory cytokines such as IL6 and TNFα (37). Macrophage reprogramming enhances paracrine communication, a process in which macrophages release mediators that directly affect the proliferation and invasiveness of tumor cells. In the present study, MDA levels were markedly higher in patients with PDAC than in the control group (38, 39). Similar observations were made in studies investigating not only pancreatic cancer, but also other gastrointestinal malignancies. Zińczuk et al. (31) reported significant differences in AGE, AOPP, and MDA levels between patients with colorectal cancer and the control group. Their findings clearly indicated that MDA levels were associated with the severity of inflammation in cancer patients. Moreover, MDA was identified as a potential predictor of tumor invasion depth and lymph node metastasis (40). The present results and the findings of other authors suggest that malignant neoplasms are associated with redox imbalance and, consequently, elevated oxidative stress parameters. Similar observations were made in studies investigating other segments of the gastrointestinal tract, which suggests that redox disturbances are a universal feature of oncogenesis (41–43).

The effects of immunomodulatory nutritional treatment on oxidative stress and the inflammatory response in pancreatic cancer have not been described to date. Available research focuses primarily on the influence of postoperative immunonutrition administered to patients with pancreatic cancer and its impact on recovery and the incidence of postoperative complications (44–46). The recommendations regarding immunonutrition are not limited specifically to patients with pancreatic cancer, but are incorporated into broader clinical nutrition guidelines for oncological surgery and Enhanced Recovery After Surgery (ERAS) perioperative care protocols, which are regularly updated by the European Society for Clinical Nutrition and Metabolism (ESPEN) and the Polish Society for Parenteral, Enteral, and Metabolic Nutrition (POLSPEN). According to these guidelines, preoperative immunonutrition should be administered orally for five to seven days in patients scheduled for pancreaticoduodenectomy, which implies that such intervention may reduce the incidence of infectious complications (47–49).

The present study showed that a seven-day immunomodulatory nutritional intervention was associated with favorable changes in selected redox balance parameters in patients with PDAC. TOS and OSI values decreased after preoperative immunonutrition, accompanied by increased TAC and FRAP levels, suggesting an improvement in redox balance and antioxidant defense capacity.

The findings of the present study suggest that preoperative immunomodulatory nutrition may contribute to reducing oxidative stress and improving redox balance before surgical treatment (50). To date, no studies have evaluated the impact of preoperative immunonutrition on oxidative stress in patients with pancreatic cancer. Bigagli et al. (51) investigated the effects of preoperative immunonutrition in patients undergoing intestinal surgery for active Crohn’s disease refractory to pharmacological treatment. In the study group, half of the patients were orally administered Impact Oral^®^ (Nestle) twice daily for seven days before surgery, similarly to the protocol applied in the present study. Serum AGE and AOPP levels were lower in the group receiving immunonutrition than in patients without nutritional intervention. In gastrointestinal malignancies, a diet rich in immunomodulatory components may increase the activity of antioxidant enzymes such as catalase, glutathione peroxidase, and superoxide dismutase (SOD), thereby enhancing antioxidant defenses against ROS (52). Arginine, one of the components of immunonutrition, is a precursor of nitric oxide (NO), which plays a key role in vascular regulation and endothelial function. Nitric oxide acts as a signaling molecule that can neutralize ROS and reduce oxidative stress. In addition, NO may influence signaling pathways that regulate the expression of antioxidant genes, which increases the production of protective enzymes (53). The Impact Oral^®^ preparation also contains omega-3 fatty acids, which may directly reduce ROS production by cancer cells. Research has shown that supplementation with EPA and DHA decreases the activity of enzymes responsible for ROS generation, such as NADPH oxidase. Omega-3 fatty acids may also increase the fluidity of cell membranes and their resistance to lipid peroxidation, thus preventing the formation of toxic products such as MDA (54). Impact Oral^®^ also contains nucleotides, which are essential for the repair of DNA damage induced by ROS. Nucleotide supplementation supports DNA repair processes and attenuates the effects of oxidative stress (55, 56).

The present study suggests that immunonutrition may exert antioxidant effects. A statistically significant decrease in AOPP and AGE concentrations, accompanied by a reduction in MDA levels, was observed after seven days of immunonutrition. A subsequent increase in protein glycoxidation and lipid peroxidation markers on postoperative day 10 appeared to align with inflammation induced by extensive tissue damage during surgery and infiltration of immune cells that triggered rapid production of cytokines and ROS. Inflammation is yet another detrimental factor that affects the development and progression of pancreatic cancer. Chronic inflammation may induce changes in the tumor microenvironment, promoting angiogenesis, proliferation of tumor cells, and their resistance to treatment (57). Neutrophils, macrophages, and myeloid-derived suppressor cells that secrete IL6, TNFα, IL1β, and transforming growth factor β (TGFβ) are recruited within the tumor microenvironment (58, 59). These cytokines activate nuclear factor kappa-light-chain-enhancer of activated B cells (NF-κB) and Signal transducer and activator of transcription 3 (STAT3) signaling pathways in tumor and stromal cells, which increases the expression of proangiogenic genes such as vascular endothelial growth factor (VEGF) and matrix metalloproteinase 9 (MMP-9) and leads to the formation of an abnormal vascular network that facilitates tumor growth and metastasis. The activated inflammatory pathways also stimulate cell proliferation and migration: IL6/STAT3 inhibits apoptosis, whereas TGF-β induces epithelial–mesenchymal transition (EMT), thus enhancing the invasiveness of cancer cells (60, 61).

In the current study, inflammatory parameters were significantly elevated in patients with PDAC, compared with the control group, as reflected by increased concentrations of all analyzed cytokines (IL1α, IL1β, IL6, IL10, and TNFα). These findings align with recent literature demonstrating that baseline systemic inflammatory and metabolic alterations at the time of diagnosis are strongly linked to clinical status and disease prognosis in pancreatic cancer (62). Pro-inflammatory cytokines such as TNFα, IL6, IL1β are produced by inflammatory cells and may stimulate the growth of tumor cells and increase their resistance to apoptosis (programmed cell death), thereby contributing to cancer progression (63). This study demonstrated that immunonutrition was associated with lower concentrations of the analyzed inflammatory cytokines during the preoperative period. However, despite the initial reduction in inflammation, a subsequent increase in IL1α, IL1β, and IL6 concentrations was observed on postoperative day 10 in patients receiving and not receiving immunonutrition. The postoperative increase in inflammatory cytokine levels is a consequence of tumor resection, which leads to a rapid surge in the production of inflammatory cytokines (IL1α, IL1β, IL6), characteristic of the flow phase of the injury response (64–66). The surgery-induced inflammatory response is highly resistant to prior immunological modulation. Even if preoperative inflammation is reduced, tissue suturing, ischemia-reperfusion injury, and the secondary immune response generate a new, strong pro-inflammatory and oxidative stimulus. Moreover, increased intake of amino acids, particularly arginine, and nucleotides enhances the proliferation of immune and connective tissue cells, which additionally increases the production of acute-phase proteins such as CRP and fibrinogen. Even the most effective preoperative nutritional intervention is unable to fully suppress the local inflammatory response induced by ischemia and reperfusion. Excessive production of ROS and cytokines at the injury site exceeds the capacity of local and systemic anti-inflammatory defense mechanisms (67, 68).

The present findings underscore the importance of appropriate preoperative preparation in patients with pancreatic cancer due to their significantly elevated levels of oxidative stress and inflammatory markers compared with those observed in the control group. Perioperative oral immunonutrition was associated with reduced oxidative stress, as reflected by lower TOS and OSI values, decreased concentrations of protein glycoxidation and lipid peroxidation markers, lower levels of inflammatory cytokines, and higher TAC values. However, the subsequent increase in biomarker levels indicates that preoperative immunonutrition does not fully alleviate the oxidative stress induced by surgical intervention. Furthermore, the present study evaluated only biochemical biomarkers and did not assess clinically meaningful outcomes. Therefore, it remains unclear whether the observed laboratory changes translate into reduced postoperative complications, improved recovery, shorter hospital stay, or enhanced quality of life. These findings suggest that preoperative immunomodulatory nutritional support may favorably influence oxidative stress and inflammatory responses in patients with pancreatic cancer; however, further prospective studies evaluating clinically relevant outcomes are required to confirm its clinical benefit.

Despite these promising findings, the study had several limitations, including a relatively small sample size and a short follow-up period. Studies with larger patient populations and longer follow-up are needed to fully evaluate the long-term benefits and potential risks associated with immunomodulatory nutritional treatment in patients with pancreatic cancer. In the future, large-scale randomized studies should be conducted to enable a more precise assessment of the efficacy of immunonutrition in this patient population. These efforts may contribute to improved survival outcomes in patients with pancreatic cancer.

A limitation of this study is that the investigator responsible for patient enrollment also generated the randomization sequence. Although a computer-generated randomization schedule was used, independent allocation concealment was not implemented, which may have increased the risk of selection bias. In addition, owing to the nature of the intervention, blinding of patients, investigators, and surgeons was not feasible. However, laboratory personnel were blinded to treatment allocation by means of coded blood samples. Despite these measures, the possibility of residual bias cannot be completely excluded.

Although most baseline demographic and clinical characteristics were comparable between groups, statistically significant differences were observed in tumor size, histological grade, and preoperative body weight loss. These baseline imbalances may have influenced postoperative outcomes and biomarker profiles independently of the nutritional intervention. Owing to the relatively small sample size, multivariable adjustment for potential confounders was not performed, as inclusion of multiple covariates could have resulted in model overfitting and unstable estimates. Therefore, the possibility of residual confounding cannot be completely excluded. Moreover, although nutritional status was assessed preoperatively using the Subjective Global Assessment (SGA), the influence of subtle differences in baseline nutritional status on postoperative outcomes and biomarker levels cannot be entirely excluded. Nutritional status is an important determinant of immune response and postoperative recovery in patients with PDAC.

Another limitation is the potential influence of perioperative and treatment-related factors on inflammatory and oxidative stress markers. The type of surgical procedure was determined by tumor location and clinical indications; therefore, different pancreatic resections were performed. Furthermore, operative time, intraoperative blood loss, blood transfusion requirements, and postoperative complications were not included in the statistical analysis. In addition, although all patients were treated at a single center according to standardized perioperative protocols, including routine use of analgesics, antibiotics, anticoagulants, and other supportive therapies as clinically indicated, the effects of concomitant medications were not analyzed separately. Therefore, residual confounding related to perioperative management and pharmacological treatment cannot be entirely excluded. Furthermore, the study evaluated only short-term laboratory biomarkers rather than postoperative clinical outcomes. Because these laboratory markers serve as surrogate endpoints, it is currently unknown whether they translate into actual clinical benefits, highlighting the need for further clinical observations and long-term follow-up.

Finally, no formal adjustment for multiple comparisons was performed. Given the number of statistical tests conducted, the risk of Type I error may have been increased, and some statistically significant findings should therefore be interpreted with caution until confirmed in larger, preferably multicenter, prospective studies.

Further prospective studies evaluating clinically relevant outcomes are required to confirm the clinical significance of these findings.

## Data Availability

The original contributions presented in the study are included in the article/supplementary material. Further inquiries can be directed to the corresponding author.
